# Kinematic Origins of Motor Inconsistency in Expert Pianists

**DOI:** 10.1371/journal.pone.0161324

**Published:** 2016-08-18

**Authors:** Kenta Tominaga, André Lee, Eckart Altenmüller, Fumio Miyazaki, Shinichi Furuya

**Affiliations:** 1 Department of Engineering Science, Osaka University, 1–3 Machikaneyama, Toyonaka, Osaka, 5608531, Japan; 2 Neurologische Klinik und Poliklinik, Klinikum rechts der Isar, Technische Universität München, Munich, Germany; 3 Institute for Music Physiology and Musicians’ Medicine, Hannover University for Music, Drama and Media, Emmichplatz 1, 30175 Hannover, Germany; 4 Musical Skill and Injury Center (MuSIC), Sophia University, Tokyo, Japan; University of Pécs Medical School, HUNGARY

## Abstract

For top performers, including athletes and musicians, even subtle inconsistencies in rhythm and force during movement production decrease the quality of performance. However, extensive training over many years beginning in childhood is unable to perfect dexterous motor performance so that it is without any error. To gain insight into the biological mechanisms underlying the subtle defects of motor actions, the present study sought to identify the kinematic origins of inconsistency of dexterous finger movements in musical performance. Seven highly-skilled pianists who have won prizes at international piano competitions played a short sequence of tones with the right hand at a predetermined tempo. Time-varying joint angles of the fingers were recorded using a custom-made data glove, and the timing and velocity of the individual keystrokes were recorded from a digital piano. Both ridge and stepwise multiple regression analyses demonstrated an association of the inter-trial variability of the inter-keystroke interval (i.e., rhythmic inconsistency) with both the rotational velocity of joints of the finger used for a keystroke (i.e., striking finger) and the movement independence between the striking and non-striking fingers. This indicates a relationship between rhythmic inconsistency in musical performance and the dynamic features of movements in not only the striking finger but also the non-striking fingers. In contrast, the inter-trial variability of the key-descending velocity (i.e., loudness inconsistency) was associated mostly with the kinematic features of the striking finger at the moment of the keystroke. Furthermore, there was no correlation between the rhythmic and loudness inconsistencies. The results suggest distinct kinematic origins of inconsistencies in rhythm and loudness in expert musical performance.

## Introduction

Motor excellence in top athletes and expert musicians typically involves the production of highly consistent motor actions. Subtle variability of movements can spoil a successful performance in sport activities and musical performance, which occurs occasionally even among expert individuals. For example, highly skilled pianists who undergo deliberate practice over years display small but non-negligible rhythmic inaccuracies in keystrokes [1,2]. Such motor variabilities are not trivial but are rather a focus of training and pedagogy for top performers. To optimize physical training and education, it is crucial to determine the specific joints that produce the variabilities of movement because skilled and naturalistic motor behaviors mostly involve motions at multiple joints. For example, in ball throwing, the variability of the ball height at the target is associated with inappropriate timing of the onset of rotation of the fingers but not with the proximal joint motions [3]. In violin playing, the amount of variability of the bow angle is correlated not with the elbow joint range of motion but with the shoulder joint range of motion [4]. These studies suggest that the trial-to-trial variability of motor performance originates from the kinematic variability of not all but some specific joints of the extremities.

In the motor system, the hand possesses a relatively large number of joints compared with the arm and leg. A particular feature of the hand is the biomechanical and physiological connection between the digits, which includes intertendinous and intermuscular connections [5,6], synchronous firing of motor neurons innervating different muscular compartments [7,8], and shared motor neurons connecting multiple digits [9]. Consequently, motion at the tip of a certain finger can result from the joint motions of not only this finger but also the adjacent fingers, so called enslavement [10,11]. Identifying the kinematic origins of the inter-trial variability of skilled digital movements such as musical performance thus requires accounting for the movement coordination between multiple joints of the digits of the hand.

Previous studies that investigated the hand kinematics of piano playing demonstrated highly individuated movements across fingers [12,13]. Typically, the middle and ring fingers are functionally less independent compared to the index and little fingers [14] due to the biomechanical constraints induced by intertendinous connections and neurophysiological constraints. In contrast, expert pianists moved all fingers equally independently [12], even when playing as fast as possible. This demonstrates how professional pianists are able to compensate for the inter-digit constraints, most likely due to prolonged practice, strengthening of the extensor muscles, and specific training of individuation. This is due to the plastic adaptation of both the peripheral biomechanical apparatus and central nervous movement representations, as shown by the fact that daily piano practicing progressively enhances the individuated finger movements in musically untrained individuals [15]. It is therefore not obvious whether the movement relationship between digits can be the kinematic origin of the spatiotemporal variability of expert musical performance. Understanding this can shed light on the biomechanical and physiological mechanisms that subserve the acquisition and production of highly dexterous motor actions [16], which is also of clinical significance because the repetition of precise movements can trigger movement disorders that cause loss of fine motor control, such as tremor [17,18] and focal dystonia [19–21].

The present study aims to identify the kinematic features associated with the inconsistency of piano performance in highly skilled pianists. As indices of the inconsistency of performance, we focused on the rhythmic variability of keystrokes and the variability of loudness of tone production across trials. The previous observation of the prominent independence of movement control across digits in expert pianists led us to postulate that the inter-trial variability of tone production can be predicted based solely on the movements and postures of the digit responsible for eliciting the tone. To test this hypothesis, stepwise multiple regression and ridge regression analyses were performed based on the kinematic features of all digits, which were measured using a custom-made data glove [22]. The predictors included the joint kinematics of the finger striking a key, as characterized by flexion of the metacarpophalangeal joint, extension of the proximal and distal phalangeal joints, and the movement covariation between the fingers.

## Materials and Methods

### Participants

Seven highly skilled adult pianists with no history of neurological disorders participated in the present experiment (21–39 years old, all right-handed, 4 females). All of the participants had won prizes at international piano competitions. In accordance with the Declaration of Helsinki, the experimental procedures were explained to all participants. Written informed consent was obtained from all participants prior to participation in the experiment, and the whole experimental protocol was approved by the ethics committee of Hannover Medical School (Approval Number: 1319–2012).

### Experimental design

**Fig 1:**
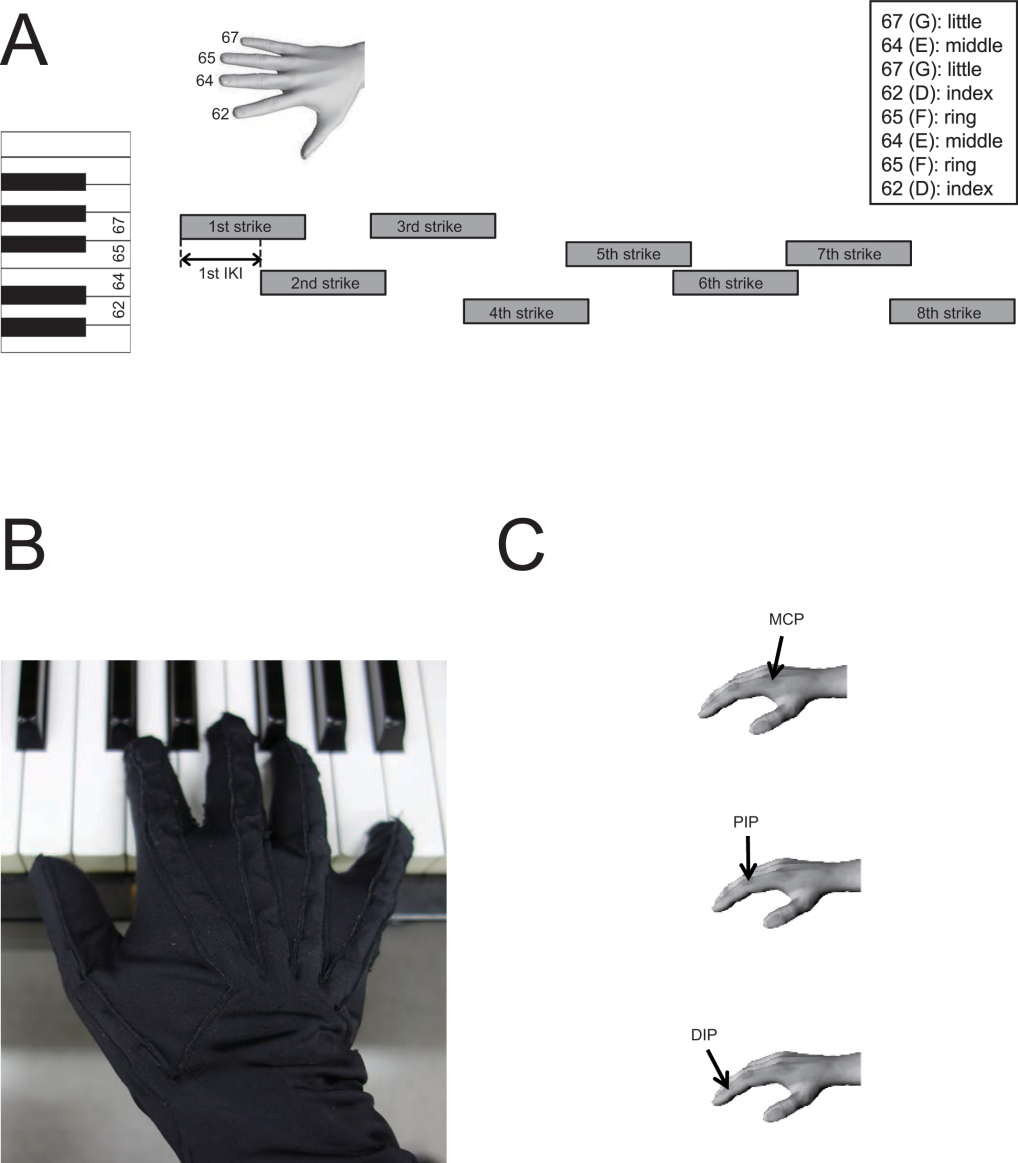
(A) The designated musical task using four fingers. The number indicates the MIDI pitch number. IKI: inter-keystroke interval. G, F, E, D: pitch notation. (B) Custom-made data glove including sensors surrounding each of the three joints of the individual fingers. (C) Joint notation: MCP, PIP, and DIP joints indicate the third, second, and first joints of each finger, respectively.

We asked participants to play a short melody requiring the use of the right hand. The melody consisted of eight notes (G-E-G-D-F-E-F-D) within a range of one octave with a specified fingering (5-3-5-2-4-3-4-2, where 2, 3, 4, and 5 represents the index, middle, ring, and little finger, respectively) (Fig 1A). A musical score with the fingering was visually presented on a computer monitor located in front of the piano, but only during the familiarization session prior to the experiment, during which a short practice period was allowed to familiarize participants with both the piano and melody. Prior to each trial, the pianists heard a recording of the target melody played with the target loudness (75 MIDI velocity, mezzo-forte) and tempo (inter-keystroke interval: IKI = 250 msec). The target melody was to be played with legato touch, meaning that a key was not released until the next key was depressed. The legato touch was instructed rather than the staccato one because it requires greater temporal coordination of movements between fingers. The hand was instructed to remain in a fixed position throughout the experiment. In total, each participant underwent 20 trials. None of the pianists struck an incorrect key throughout the experiment. The pianists played a digital piano with a mechanical action similar to that of an acoustic piano (MP 9000, KAWAI, Krefeld, Germany). The design of the present study allowed us to obtain between-trial and within-finger-transition variabilities of the rhythm and loudness of a performance for the regression analysis (mentioned in Data Analysis and Statistics), which matters even for highly skilled pianists.

### Data acquisition

We recorded the time-varying dynamic changes in the pianists’ finger joint angles using sensors embedded in a right-handed custom-made glove [22] (Fig 1B). The glove was thin, flexible and open at the fingertips. We recorded the motions at 12 joints at 1-msec intervals (i.e., sampling frequency = 1 kHz). The measured angles were the metacarpo-phalangeal (MCP), proximal-phalangeal (PIP), and distal-phalangeal (DIP) joint angles of the four fingers (Fig 1C). We did not record the thumb joint angles due to a lack of a sensor that accurately measures the angle of thumb rotation about an axis passing through the trapeziometacarpal joint of the thumb and index MCP joint in this glove. We therefore chose a task that requires movement of the remaining four fingers. Extension was defined as positive; the angles were defined as 0 when the finger was straight and in the plane of the palm.

We also recorded MIDI data from the keyboard using a custom-made script in LabVIEW (National Instruments, Texas, United States), running at 1 kHz in synchronization with the data glove. From the MIDI data, we derived the velocity with which each key was depressed (i.e., key-striking velocity as an index of “loudness”) and the time each key was depressed (i.e. MIDIon) and released (i.e. MIDIoff).

### Data analysis and statistics

Using the MIDI information, we evaluated the spatiotemporal accuracy of finger movements by computing the inter-trial variability of (1) the interval between two successive keystrokes as an index of tempo (i.e., inter-keystroke interval: IKI) and (2) the key-striking velocity as an index of loudness (i.e., MIDI velocity) for individual strokes (i.e., 8 strikes) across trials for each participant. Here, the IKI was defined as an interval between the time-points of MIDIon of two successive keystrokes.

To describe the features of joint kinematics of the finger involved in the individual piano keystrokes, the angles and angular velocities of the MCP, PIP, and DIP joints of the finger used for the keystroke (“striking finger”) were computed at the moment of note onset, defined by MIDIon, for each of twenty trials. To assess the movement independence between the fingers during the individual keystrokes, a correlation coefficient of the joint angular movements between the striking finger and each of the other fingers was computed at each of the three joints during the period between the moment of the keystroke by the striking finger and its prior keystroke for each of the twenty trials. For example, for the initial IKI between the little finger keypress and the middle finger keypress, the striking finger was the middle finger (i.e., the latter of the two successive keystrokes); therefore, a correlation coefficient was computed between the middle finger and each of the three remaining fingers during this IKI.

To account for the inter-trial variability of each MIDI variable (i.e., motor performance) according to the kinematics of the finger joint, a stepwise multiple regression analysis was performed. First, each of the kinematic variables was standardized for each participant, which consisted of dividing by one standard deviation across the trials after subtracting the mean value. Second, using the standardized values of each kinematic variable for all participants as independent variables, a stepwise multiple regression analysis was run, in which the dependent variable was the standardized values of each MIDI variable for all participants (20 trials x 7 participants = 140 datapoints). Here, we used a bidirectional stepwise regression to circumvent converging to the local minimum. The analysis was carried out using MATLAB (Mathworks Co.), and significance was set at p < 0.05. However, the stepwise multiple regression could yield erratic results if there was a correlation between the independent variables, a so-called multicollinearity problem. To evaluate the validity of the derived results, we also performed a ridge multiple linear regression with L2 norm regularization, which is an analysis to cope with multicollinearity. Here, the partial regression coefficients were estimated according to cross validation.

## Results

### Inter-trial variability of the IKI and key-striking velocity

The group mean of the inter-trial variability of the IKI was 7.9 ± 1.2 ms, which ranged from 4.5 to 12.3 ms at the individual strike level. The group mean of the inter-trial variability of the key-striking velocity was 4.1 ± 1.2 MIDI velocity, which ranged from 1.2 to 7.5 MIDI velocity at the individual strike level. To assess whether the rhythmic variability was correlated with the variability of key-striking velocity, a correlation analysis was performed between these variables. The correlation coefficient and p value were 0.08 and 0.32, respectively, which nullified their correlation.

### Joint kinematics

**Fig 2:**
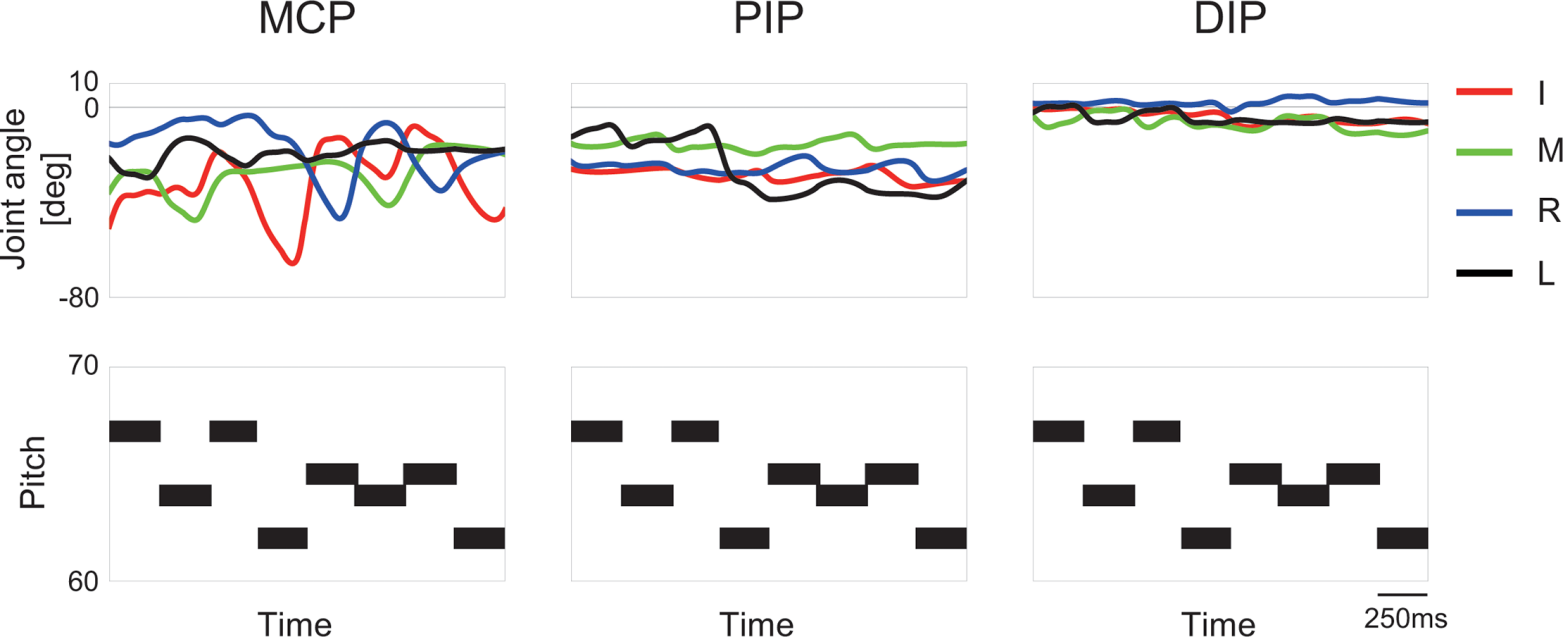
**The time-varying angular positions of the MCP, PIP, and DIP joints of the index, middle, ring, and little fingers (upper panel) and time-synchronized MIDI datasets representing the moments of tone onset (MIDIon) and offset (MIDIoff) at each of eight keypresses**. For the upper panel, the y-axis is a joint angle of the fingers in degree.

Fig 2 illustrates the time-varying angular positions of the MCP, PIP, and DIP joints of the index, middle, ring, and little fingers and MIDI information representing the moments the finger touched and released the key at each of eight keypresses within a single trial in one representative pianist. The angular data was low-pass filtered using a Butterworth filter with a cutoff frequency of 24Hz. While striking a key, the MCP and PIP joints of the striking finger were rotating for flexion and extension, which changed its direction while the finger was contacting a key.

**Fig 3:**
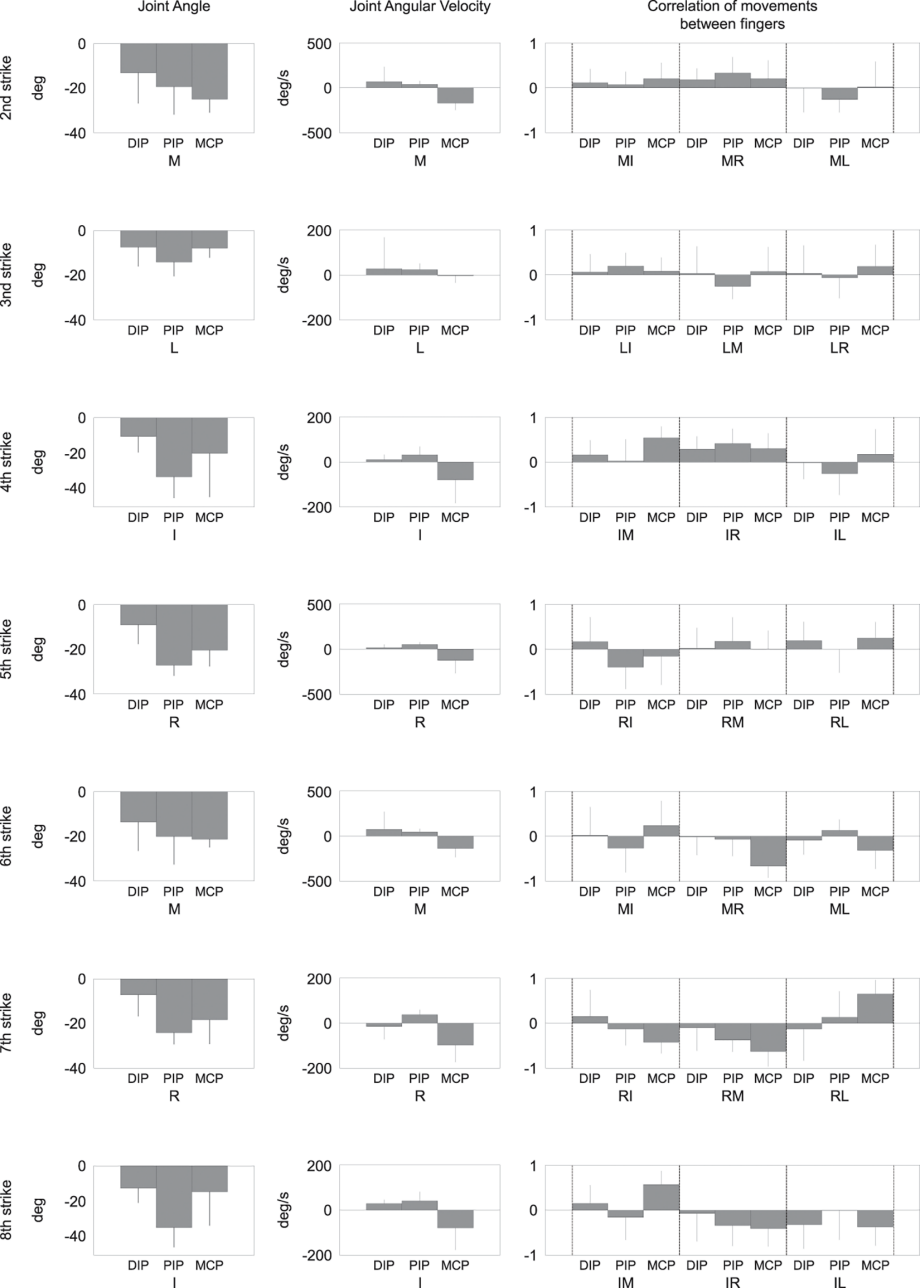
Group means of the individual kinematic variables at the individual strikes. I, M, R, and L indicate the index, middle, ring, and little fingers, respectively. The error bar indicates one standard deviation. Left panel: joint angle of the striking finger, Middle panel: joint angular velocity of the striking finger, Right panel: correlation coefficients of movement between the striking finger and each of the remaining fingers. For example, the 2^nd^ strike was made by the middle finger, which is defined as the striking finger. Here, a correlation was computed during the period between the 1^st^ and 2^nd^ strikes.

Fig 3 illustrates the group means of the individual kinematic variables including the joint angle and joint angular velocity of the striking finger at the moment of the individual strikes, and the correlation coefficients of movements between the striking finger and the other non-striking fingers. The angles of the MCP, PIP, and DIP joints were all negative irrespective of the striking finger, which indicates that joints of the striking finger were flexed at the moment of a keypress. The angular velocity was overall positive at the DIP and PIP joints (i.e., extension) and all negative at the MCP joint (i.e., flexion) at the moment of a keypress by the target finger. The correlation coefficient of movements between the fingers at each joint varied depending on which finger was used for the strike. From the second to fourth strikes, almost all coefficients between the striking and non-striking fingers were positive, which indicated simultaneous movements at a pair of fingers in the same direction. The sign turned to the opposite from the sixth to eighth keystrokes, although the striking finger and its adjacent finger displayed a positive correlation at the MCP joint (i.e., RL at the 7^th^ strike and IM at the 8^th^ strike).

### Variability of the IKI across trials (rhythmic inconsistency)

Table 1 summarizes the results of a stepwise regression analysis to predict the inter-trial variability of the IKI according to the finger joint kinematics. Overall, two predominating kinematic variables were significantly associated with the rhythmic inconsistency of keystrokes across trials: (1) the joint angle and angular velocity of the striking finger at the moment of the keypress and (2) the movement independence between the striking finger and the adjacent finger. For the first IKI of the target melody (i.e., the interval between the first and second keystrokes with the little and middle fingers), significant predictors of the rhythmic inconsistency were confined to the MCP and PIP joint velocities of the middle finger used for the latter of two successive keystrokes. For the second IKI with the middle and little fingers, the regression analysis identified both the MCP joint angle of the little finger at the moment of the keypress with this finger and a correlation coefficient of the PIP joint angular velocity between the little and index fingers. For the third IKI with the little and index fingers, not only the PIP joint angular velocity of the index finger but also the correlation coefficients of the angular velocity at the MCP joint between the index (striking finger) and middle fingers and at the PIP joint between the index and little fingers served as significant predictors of the inter-trial variability. There was no significant predictor of the fourth IKI. Concerning the fifth and sixth IKIs, the joint angular velocities of the middle MCP and ring DIP were significant predictors. Finally, a significant predictor of the seventh IKI between the ring and index finger keystrokes was a correlation coefficient of the angular velocity between the index and middle fingers.

**Table 1:** Results of stepwise multiple regression predicting the inter-trial variability of the individual inter-keystroke intervals (IKIs).

		Kinematics of the striking finger at the moment of a keypress						Correlation coefficient of the finger movements during two successive keystrokes								
								between a striking finger and the first finger from the radial			between a striking finger and the second finger from the radial			between a striking finger and the third finger from the radial		
		Joint angle			Joint angular velocity			Joint angular velocity			Joint angular velocity			Joint angular velocity		
		DIP	PIP	MCP	DIP	PIP	MCP	DIP	PIP	MCP	DIP	PIP	MCP	DIP	PIP	MCP
First IKI	coef.	0.072	0.020	-0.127	0.083	**0.181**	**-0.210**	0.030	-0.107	-0.125	-0.088	-0.051	-0.109	0.082	-0.064	-0.041
	p	0.386	0.817	0.122	0.381	**0.030**	**0.012**	0.720	0.193	0.142	0.292	0.535	0.182	0.327	0.483	0.620
Second IKI	coef.	-0.144	-0.036	**0.216**	0.065	-0.005	0.003	0.130	**0.183**	-0.097	-0.115	-0.020	-0.057	0.001	0.043	0.003
	p	0.087	0.699	**0.010**	0.433	0.952	0.975	0.115	**0.028**	0.244	0.170	0.812	0.493	0.989	0.620	0.968
Third IKI	coef.	-0.093	0.099	0.018	-0.056	**0.231**	0.002	-0.146	-0.034	**-0.257**	-0.144	-0.079	-0.038	0.074	**0.326**	0.074
	p	0.254	0.217	0.822	0.494	**0.009**	0.976	0.092	0.687	**0.002**	0.075	0.348	0.649	0.365	**0.000**	0.375
Fourth IKI	coef.	-0.028	-0.074	-0.089	0.033	0.098	-0.082	0.115	-0.079	0.015	0.088	0.015	-0.008	0.094	-0.006	0.052
	p	0.747	0.385	0.293	0.696	0.249	0.333	0.176	0.354	0.863	0.301	0.862	0.926	0.271	0.948	0.544
Fifth IKI	coef.	-0.090	-0.016	0.143	0.045	0.071	**-0.252**	0.096	0.046	0.080	-0.054	-0.013	0.146	-0.075	-0.033	0.053
	p	0.275	0.851	0.084	0.591	0.400	**0.003**	0.251	0.575	0.337	0.513	0.876	0.091	0.371	0.693	0.523
Sixth IKI	coef.	-0.058	0.159	0.030	**0.202**	-0.139	-0.102	0.083	0.099	0.112	-0.025	-0.016	0.123	-0.016	0.089	0.092
	p	0.508	0.057	0.723	**0.017**	0.096	0.229	0.326	0.241	0.186	0.763	0.849	0.152	0.853	0.294	0.282
Seventh IKI	coef.	0.008	-0.103	-0.031	0.131	0.147	-0.009	-0.093	-0.024	**-0.233**	0.099	0.003	0.001	0.153	0.028	-0.029
	p	0.922	0.224	0.719	0.115	0.076	0.913	0.269	0.771	**0.006**	0.242	0.971	0.993	0.064	0.738	0.724

A number in bold indicates p < 0.05.

### Variability of the key striking velocity across trials (loudness inconsistency)

Table 2 summarizes the results of a stepwise regression analysis predicting the inter-trial variability of the key striking velocity (i.e., tone loudness). In most cases, the joint angular velocity and/or joint angle of the striking finger were significant predictors of tone loudness, whereas the inter-finger movement independence was not, except for the final keystroke. Here, loudness inconsistency was also significantly associated with the correlation coefficients of movements between the index and little fingers at the PIP and MCP joints.

**Table 2:** Results of a stepwise multiple regression predicting the MIDI velocity of the individual keystrokes.

		Kinematics of the striking finger at the moment of a keypress						Correlation coefficient of the finger movements during two successive keystrokes								
								between a striking finger and the first finger from the radial			between a striking finger and the second finger from the radial			between a striking finger and the third finger from the radial		
		Joint angle			Joint angular velocity			Joint angular velocity			Joint angular velocity			Joint angular velocity		
		DIP	PIP	MCP	DIP	PIP	MCP	DIP	PIP	MCP	DIP	PIP	MCP	DIP	PIP	MCP
Secondstrike	coef.	0.035	-0.077	-0.062	0.122	-0.027	**-0.302**	0.127	0.000	-0.086	-0.010	-0.040	0.016	0.013	-0.054	-0.009
	p	0.665	0.344	0.446	0.146	0.742	**0.000**	0.125	0.996	0.310	0.901	0.626	0.840	0.871	0.506	0.913
Third strike	coef.	**-0.201**	0.069	-0.009	-0.145	0.051	0.091	-0.030	0.145	0.001	-0.124	0.050	0.024	-0.004	0.038	0.046
	p	**0.017**	0.434	0.918	0.103	0.547	0.284	0.723	0.086	0.994	0.142	0.555	0.778	0.966	0.653	0.587
Fourth strike	coef.	0.094	-0.030	-0.055	**-0.171**	0.017	0.058	0.085	0.083	0.089	-0.071	0.088	-0.001	0.015	0.122	0.107
	p	0.273	0.727	0.512	**0.044**	0.841	0.517	0.312	0.328	0.292	0.402	0.294	0.988	0.861	0.155	0.206
Fifth strike	coef.	-0.117	-0.064	0.126	-0.016	**-0.262**	**-0.214**	0.058	-0.161	-0.043	-0.092	0.013	-0.034	0.009	0.042	-0.057
	p	0.154	0.437	0.179	0.849	**0.002**	**0.011**	0.477	0.073	0.619	0.274	0.871	0.688	0.910	0.620	0.486
Sixth strike	coef.	0.004	**-0.187**	0.099	0.000	-0.006	**-0.228**	-0.020	-0.003	0.088	0.020	0.010	0.093	0.011	0.105	0.070
	p	0.965	**0.024**	0.232	0.996	0.944	**0.006**	0.805	0.967	0.292	0.813	0.903	0.280	0.900	0.206	0.400
Seventh strike	coef.	0.022	-0.022	0.002	0.082	-0.109	0.016	0.022	-0.003	-0.098	-0.036	0.007	0.092	0.066	0.022	-0.137
	p	0.793	0.800	0.985	0.338	0.202	0.849	0.800	0.968	0.251	0.674	0.938	0.278	0.438	0.793	0.107
Eighth strike	coef.	**0.244**	0.074	0.123	-0.120	-0.079	-0.047	-0.075	0.090	-0.076	0.154	0.056	0.051	-0.063	**-0.173**	**-0.188**
	p	**0.003**	0.465	0.138	0.141	0.329	0.572	0.348	0.273	0.358	0.055	0.539	0.536	0.440	**0.034**	**0.022**

A number in bold indicates p < 0.05.

Here, the first strike was not analyzed because the movement correlation was computed during a period between the target strike and its preceding strike.

### Results of ridge regression

Table 3 summarizes the partial regression coefficients of the individual kinematic predictors when predicting the inter-trial variability of IKI and key striking velocity. Concerning the prediction of the IKI variability, most of the significant predictors determined by the stepwise regression displayed a large coefficient value with the same sign (e.g., angular velocity of the PIP and MCP joints at the 1^st^ IKI). However, a few variables did not show a large coefficient value, though the stepwise regression identified them as significant (e.g., the MCP joint angular velocity at the 5^th^ IKI). Concerning the prediction of the MIDI velocity, some of the significant predictors determined by the stepwise regression displayed a large coefficient value in the ridge regression (e.g., DIP joint angle at the 3^rd^ strike and DIP joint angular velocity at the 4^th^ strike). However, several variables showed small coefficient values even though the stepwise regression determined them to be significant. In addition, particularly at the 4^th^ strike, several variables exhibited a large value, which were not significant with the stepwise regression.

**Table 3:** Results of ridge regression predicting the inter-keystroke interval (IKI) and MIDI velocity (vel.) of the individual keystrokes.

	Kinematics of the striking finger at the moment of a keypress						Correlation coefficient of the finger movements during two successive keystrokes								
							between a striking finger and the first finger from the radial			between a striking finger and the second finger from the radial			between a striking finger and the third finger from the radial		
	Joint angle			Joint angular velocity			Joint angular velocity			Joint angular velocity			Joint angular velocity		
	DIP	PIP	MCP	DIP	PIP	MCP	DIP	PIP	MCP	DIP	PIP	MCP	DIP	PIP	MCP
1st IKI	0.03	-0.01	-0.05	0.05	**0.07**	**-0.08**	0.02	-0.05	-0.04	-0.02	-0.01	-0.04	0.04	-0.04	-4.0E-03
2nd IKI	**-0.06**	0.01	**0.09**	0.04	-0.01	-0.02	0.05	**0.06**	-0.05	-0.05	-0.01	-0.01	-3.3E-04	0.03	4.4E-03
3rd IKI	**-0.14**	**0.10**	-1.6E-03	-0.03	**0.13**	9.4E-04	-0.05	-0.02	**-0.14**	**-0.07**	-0.02	-0.05	0.05	**0.20**	0.05
4th IKI	-2.8E-38	-7.5E-38	-9.0E-38	3.4E-38	9.9E-38	-8.3E-38	1.2E-37	-8.0E-38	1.5E-38	8.9E-38	1.5E-38	-8.0E-39	9.5E-38	-5.7E-39	5.2E-38
5th IKI	-0.01	1.6E-04	0.02	0.01	0.02	-0.04	0.02	0.01	0.02	-0.01	3.7E-06	0.01	-0.02	-8.2E-04	0.01
6th IKI	-0.01	0.05	4.0E-05	**0.07**	-0.05	-0.05	0.02	0.04	0.05	-0.01	-4.5E-03	0.05	0.01	0.02	0.03
7th IKI	0.01	-0.03	-0.02	0.04	0.05	-0.01	-0.03	-0.01	**-0.07**	0.04	0.01	0.01	0.05	0.01	-4.7E-03
2nd strike vel.	0.02	-0.03	-0.02	0.05	-0.01	**-0.09**	0.05	-2.3E-03	-0.02	4.4E-03	-0.02	0.01	0.01	-0.02	4.1E-04
3rd strike vel.	**-0.06**	0.02	-0.01	**-0.07**	0.02	0.04	-0.01	0.05	-1.2E-03	-0.05	0.01	0.01	3.5E-03	0.01	0.02
4th strike vel.	**0.07**	-0.03	-0.05	**-0.10**	0.01	0.05	0.04	**0.08**	0.05	-0.05	0.05	-0.02	0.03	**0.10**	0.05
5th strike vel.	-1.0E-03	-3.6E-04	9.0E-04	-1.4E-04	-2.3E-03	-1.7E-03	6.0E-04	-9.8E-04	-1.1E-03	-3.7E-04	-2.0E-04	3.5E-04	3.9E-04	-1.4E-04	-4.8E-04
6th strike vel.	-4.9E-03	-0.01	4.4E-03	0.01	0.01	-0.02	4.8E-04	2.5E-05	0.01	-3.9E-04	9.9E-04	8.3E-04	-3.1E-03	0.01	0.01
7th strike vel.	0.01	-0.01	-7.7E-04	0.03	-0.05	-8.7E-04	0.01	-1.7E-03	-0.05	-0.02	-3.7E-03	0.04	0.03	0.01	-0.05
8th strike vel.	**0.06**	0.03	0.02	-0.02	-0.01	-0.03	-0.02	0.01	-0.02	0.04	-0.01	0.01	-0.01	-0.04	-0.04

A value in bold indicates that the absolute value is larger than 0.06

An underlined value indicates the variable in which the stepwise regression is significant. (see Tables 1 and 2)

E-n indicates x 10-n

### Accuracy of tempo

To assess the accuracy of tempo within a trial, we computed the absolute error of IKI by subtracting the ideal IKI (= 250 ms) from the observed value, which were averaged across trials and participants. The group mean of the absolute error of IKI was 8.46 ± 2.70 ms (mean ± SD), which amounted to 3.4% of the target IKI. One-way repeated measures ANOVA using the finger-transition as an independent variable (i.e. 7 levels) yielded no significant main effect of finger-transition (p > 0.05).

## Discussion

Even expert pianists who have won prizes at international piano competitions display inconsistencies in motor performance while performing precisely timed piano keystrokes. The trial-by-trial inconsistency of the keystrokes is ideally minimized to accomplish the desired musical performance at a high level of precision, which ranged from 4.5 to 12.3 ms in the present expert piano performances. Our findings are consistent with previous observations of expert pianists who displayed approximately 8 ms of rhythmic inconsistency of piano keystrokes on average [23]. However, the perceptual threshold of detectability in timing differences by experienced listeners is 4% of the interval of tones [24], which amounts to 10 ms in the present task (i.e., IKI = 250 ms). The observed rhythmic inconsistency is therefore discernible in some cases and should be kept below the threshold for pianists to accomplish the desired musical performance flawlessly.

This study extended the previous findings in terms of identifying the kinematic landmarks of the rhythmic inter-trial variability based on multivariate analysis of kinematic features of the finger movements derived from a custom-made data-glove. The results demonstrated two predominant factors linked to the rhythmic inconsistency of successive piano keystrokes by expert pianists. First, the IKI inconsistency was correlated negatively with the MCP joint velocity and positively with the PIP and DIP joint velocities (Table 1). At the moment of a keypress, the MCP joint of the striking finger was rotating in the direction of flexion, and vice versa for the PIP and DIP joints (Fig 3). Accordingly, a shorter interval of two successive keystrokes was associated with smaller rotational velocities for the MCP flexion and PIP and DIP extension of the finger responsible for the latter of the two strikes. Because these joint rotations accelerate toward the moment when the fingertip impacts with the key, a shorter IKI can result in a shorter duration of angular acceleration before the joint reaches its maximum velocity, which possibly yields slower joint rotation at the impact moment. The inconsistency of the joint rotational velocity may originate from signal-dependent noise contaminated into efferent motor commands elicited from the nervous system to the individual muscles [25–27] and/or from uncertainty of afferent sensory feedback that plays a role in maintaining movement consistency [28,29]. Our finding of the inter-trial movement inconsistency in expert pianists suggests that extensive musical training from childhood is incapable of taming the effects of the biological noise in the sensorimotor system thoroughly.

Second, the degree of movement independence between the fingers was significantly related to the degree of rhythmic inconsistency. Although expert pianists move the fingers in a more individuated manner than musically untrained individuals [12], the independent control of finger movements requires overcoming innate neurophysiological and biomechanical natures of the hand, which include anatomical inter-tendon and inter-muscular connections in the hand [5], innervation of the peripheral nerve to different finger muscles [7,8], and functional and anatomical coupling of the motor neuron innervating the finger muscles [9]. Although extensive training can bring about neuroplastic adaptation that enhances the individuated finger movements, the innate anatomical architecture is most likely incapable of adapting to thoroughly overcome the inter-digit constraints, even through long-term extensive training from childhood [30]. This fact may explain an association between the rhythmic inconsistency of musical performance and the inter-digit independence of movements. To the best of our knowledge, this association was directly demonstrated for the first time in this study, and may explain the functional role of superior independent movement control between fingers in pianists relative to musically untrained individuals.

In the present study, pianists were instructed to play with legato touch. This touch constrains the temporal relationship between the key releasing movements and subsequent key striking movements. The contribution of the movement independence between the fingers to rhythmic inconsistency of the keystrokes can be due to this specific touch. This postulation predicts that the movement independence between the striking finger and its previous striking finger is a determinant of the rhythmic inconsistency, which was however not evident in the present results (i.e., Tables 1 and 3). This touch also involves precisely timed transitions from dynamic movements to depress the key to isometric force production to keep the key depressed, which can be another potential cause of timing variability [31].

In contrast to the rhythmic variability, the inconsistency of tone loudness was, in most cases, not associated with the movement independence between the striking and non-striking fingers. Rather, we identified the association with the characteristics of movements by the striking finger. At the moment of the finger-key contact, both the force and stiffness at the fingertip depend on the geometrical configuration of the striking finger (i.e., finger posture) [32]. It is therefore reasonable that the inter-trial variability of joint angle was associated with the inconsistency of tone loudness. In addition, larger flexion velocity at the MCP joint was associated with larger tone loudness, which suggests that the erroneous control of the joint rotational speed may result in the production of a tone of unwanted loudness.

Several studies have attempted to assess the kinematic origins of the rhythmic inaccuracy of movements in musical performance [4,17,33–38]. A particular focus on the inter-trial variability was provided by a recent motion-capture study, which demonstrated a close relationship between the inter-joint movement coordination of a single finger involved in striking a key and the rhythmic inconsistency of piano performance [35]. A novelty of the present study compared to previous studies resides in identifying some particular kinematic features associated with the inconsistency of rhythm and force in musical performance by expert musicians, which, to our best knowledge, has been never investigated. An implication of the present findings may be to provide both musicians and musical teachers with clues to discern the sources of erroneous keystrokes during practicing and teaching. Due to a large number of degrees of freedom involved the in production of finger movements, pianists constantly face an ill-posed problem assigning motor error at the fingertip to joint movements and muscular activities. This requires pianists to spend a lot of time and effort in exploring and correcting the specific movements directly linked to the error. An attempt to discover the kinematic and muscular origins of performance inconsistency can therefore aid in minimizing such exploration.

Several limitations should be addressed. First, the present study did not cover all possible patterns of fingering, such as the transitions between the index and middle fingers. Because the movement independence between the fingers depends on fingering [12], the present finding cannot be extended to playing these fingering patterns in a strict sense. Second, the present study investigated a single tempo. A previous study suggested that the relationship between rhythmic inconsistencies and kinematics may change with tempo [39], whereas some studies illustrated the robustness of the timing variability of keystrokes and joint kinematics against tempo [13,35]. Third, as indices of fine motor control, we only assessed key-striking movements. Because pianists played with a legato touch requiring precise control of timing of lifting a finger from the key, it is worthwhile to identify the kinematic features associated with the precise control of the key release movements. The present study, however, did not address this because the timing of the key striking and key releasing movements typically correlate during piano performance [40]. Furthermore, as an index of the rhythmic inconsistency of the keystrokes, we computed the variability of IKI, which can be too simplistic relative to several elaborated models decomposing the temporal variability of repetitive finger tapping into components of biological significance [2,41]. We did not adopt these models due to a lack of specific and detailed hypotheses linking to them. Fourth, although the results of the stepwise and ridge regression analyses were similar, there were also some discrepancies in the results between these analyses. A selection of a smaller number of potential predictors based on specific hypotheses will be needed in future studies rather than exploratory analyses. Finally, other predictors of inconsistency of the IKI and striking velocity may remain. For example, there were no significant predictors of the key striking velocity of the fifth strike. Possible candidate predictors include the finger muscular stiffness and kinematics of the proximal joints, such as the wrist, elbow, and shoulder.
